# From structure to therapy: two novel bacteriophages from swine wastewater with divergent genomes converge in combating *Escherichia coli* and *Salmonella* infections

**DOI:** 10.3389/fmicb.2026.1763662

**Published:** 2026-02-16

**Authors:** Liyuan Zhang, Yuqi Zhou, Di Li, Xiaojing Zhuo, Shuang Li, Shaoshuai Ma, Ziyi Zhang, Wenqi Su, Ting Rong Luo, Hongyun Zhang, Jingjing Liang, Xiaoning Li

**Affiliations:** 1 College of Animal Sciences and Technology, Guangxi University, Nanning, Guangxi, China; 2 State Key Laboratory for Conservation and Utilization of Subtropical Agro-Bioresources, Guangxi University, Nanning, Guangxi, China; 3 Bama County Center for Animal Disease Prevention and Control, Hechi, China; 4 Guangxi Zhuang Autonomous Region Engineering Research Center of Veterinary Biologics, Nanning, China; 5 Guangxi Key Laboratory of Animal Breeding, Disease Control and Prevention, Nanning, China

**Keywords:** bacteriophage therapy, environmental microbiology, *Escherichia coli*, *Microviridae*, *Myoviridae*

## Abstract

Swine wastewater, characterized by a high bacterial density and significant pressure on antibiotic selection, serves as a distinct reservoir for various bacteriophages. In this study, we report the simultaneous isolation and identification of two novel bacteriophages, vB_EcoM_BYEP01 and vB_SalS_SP14, from a single pig wastewater sample. These phages specifically target original host strain *Escherichia coli* BYEC01 and *Salmonella enterica* ATCC 14028, respectively. Both phages exhibit activity within the temperature range of 30–50 °C and remain stable over a pH range of 6–10. Transmission electron microscopy revealed that vB_EcoM_BYEP01 belongs to the *Myoviridae* family and vB_SalS_SP14 to *Microviridae*. Whole-genome sequencing and comparative analyses further confirmed these phylogenetic differences. Notably, despite these significant structural and genomic differences, both bacteriophages demonstrated efficient lytic life cycles. They lack genes associated with toxins or antibiotic resistance, while demonstrating biological safety. They share key functional characteristics, including rapid adsorption rates, short incubation periods, and large outbreak sizes, highlighting their efficacy as potent bacterial killers. Both bacteriophages can be combined with antibiotics to enhance antibacterial activity and demonstrate protective effects in food (such as milk and pig skin) and mouse infection models. The coexistence of these unrelated yet highly effective bacteriophages within a single microenvironment highlights the inherent functional redundancy and evolutionary adaptability of bacteriophages. This study provides valuable insights for developing targeted phage cocktail therapies against multiple bacterial pathogens.

## 1 Introduction

Bacteriophages (phages) consist of a single type of genetic material, DNA or RNA. Similar to conventional viruses, this genetic material is enclosed within a protein capsid, collectively constituting an abiotic entity (Salmond and Fineran, 2015; Dion et al., 2020). Recently, the growing crisis of antimicrobial resistance (AMR) has sparked significant interest in phages as promising alternatives or adjuncts to traditional antibiotics in the field of phage therapy (Luong et al., 2020; Cisek et al., 2017; Rehman et al., 2019; Lin et al., 2017). This is particularly relevant in agricultural settings, such as swine production, where excessive antibiotic use has led to the emergence of multidrug-resistant pathogens, including *Escherichia coli* (*E. coli*) and *Salmonella enterica* (*S. enterica*). These pathogens pose significant threats to animal welfare and public health (Brook and Bannister, 1993; Johnston, 2000; Aarestrup et al., 2008; Han et al., 2024; Knodler and Elfenbein, 2019). Given the narrow host range of bacteriophages, it is necessary to investigate additional bacteriophages to cope with the ongoing evolution of drug-resistant bacteria.

It is essential to recognize that numerous strains of *E. coli* are harmless and can naturally coexist within the intestines (Leimbach et al., 2013). However, *Diarrheagenic E. coli* pathotypes, such as *enterotoxigenic E. coli* (ETEC) and *Shiga toxin-producing E. coli*, remain a significant global public health issue, resulting in significant mortality and morbidity, particularly in children and in animal husbandry, such as weaned piglets. The primary virulence factors of ETEC, heat-stable (ST) and heat-labile (LT) enterotoxins, trigger excessive fluid secretion in the intestine. For instance, ST binds to guanylate cyclase 2C, leading to increased intracellular cyclic guanosine monophosphate (cGMP) levels and subsequent diarrhea (Barros et al., 2025; Ramananda et al., 2025; Cho et al., 2025). *Salmonella enterica* subsp. *enterica* serovar Typhimurium ATCC 14028 (*S. enterica* ATCC 14028) is a widely utilized model organism for the study of non-typhoidal salmonellosis, owing to its well-defined genetic background and pronounced virulence in murine infection models (Branchu et al., 2018). Central to its pathogenicity are two Type III Secretion Systems (T3SS-1 and T3SS-2), encoded by Salmonella Pathogenicity Islands 1 and 2 (SPI-1 and SPI-2), respectively. T3SS-1 facilitates the initial invasion of non-phagocytic intestinal epithelial cells through the translocation of effector proteins that induce actin cytoskeletal rearrangements. Subsequently, T3SS-2 plays an essential role in bacterial survival and replication within the hostile environment of the Salmonella-containing vacuole (SCV) in host macrophages (Lou et al., 2019; Figueira and Holden, 2012). Additionally, this strain carries a virulence plasmid that contributes to systemic dissemination. Functional analyses have revealed that this plasmid is both conserved and interchangeable among common laboratory strains, including ATCC 14028, LT2, and SL1344, highlighting its broad relevance in experimental pathogenesis studies (García-Quintanilla and Casadesús, 2011).

The isolation and identification of novel bacteriophages provides a fundamental basis for advancing their application and basic research. Although numerous studies have demonstrated the efficacy of individual bacteriophages against *E. coli* or *Salmonella*, there is growing recognition that complex bacterial communities in natural environments are regulated by consortia composed of multiple bacteriophages (Stern and Sorek, 2011; Koskella and Brockhurst, 2014). In this study, we isolated and identified two novel phages from pig wastewater. Genomic analysis confirmed the absence of toxic or antibiotic-resistance genes. Notably, combining these phages with conventional antibiotics enhanced bacterial killing and suppressed regrowth. They also showed strong practical potential by significantly reducing pathogens on contaminated food surfaces and lowering mortality in mice with lethal systemic infections.

This work advances our understanding of bacteriophage diversity and evolution in complex environments, and offers promising candidates for targeted phage cocktail therapy against two major bacterial pathogens.

## 2 Materials and methods

### 2.1 Bacterial strains and growth conditions

This study used 26 bacterial strains, including 22 *E. coli* strains and 4 *Salmonella* strains, all isolated and identified by the Laboratory of Pathogenic Mechanisms and Control of Subtropical Livestock Diseases at Guangxi University or obtained through procurement (Table 1). The original bacterial strains were preserved at −80 °C. All bacteria were cultured in Luria-Bertani (LB) broth supplemented with 0.9% (w/v) NaCl at 37 °C.

**Table 1:** Bacterial strains used for host range test.

Bacterial species	Strains	Lysis	Source
*Escherichia coli*	BYEC01	1	Swine wastewater
	BYEC02	1	Swine wastewater
	BYEC03	0	Swine wastewater
	BYEC04	1	Swine wastewater
	BYEC05	1	Swine wastewater
	BYEC06	1	Swine wastewater
	BYEC07	1	Swine wastewater
	SCEC-Z02	0	Swine wastewater
	GXEC-N01	0	Swine wastewater
	GXEC-N04	0	Swine wastewater
	GXEC-N05	0	Swine wastewater
	GXEC-N06	0	Swine wastewater
	GXEC-N07	0	Swine wastewater
	GXEC-N11	0	Swine wastewater
	GDEC-F04	0	Swine wastewater
	GDEC-F05	0	Swine wastewater
	GDEC-F06	0	Swine wastewater
	GDEC-F07	0	Swine wastewater
	GDEC-F08	0	Swine wastewater
	GDEC-F11	0	Swine wastewater
	GDEC-F13	0	Swine wastewater
	CVCC 1527	0	CVCC
*Salmonella*	CVCC 3384	0	CVCC
	CVCC 1806	0	CVCC
	ATCC 14028	1	ATCC
	CMCC 50746	0	CMCC

This table shows a list of bacterial strains tested for host range test. 1, complete clearing; 0, no clearing.

### 2.2 Phage isolation and purification

The two bacteriophages, vB_EcoM_BYEP01 (infecting *E. coli* BYEC01) and vB_SalS_SP14 (infecting *S. enterica* ATCC 14028), were independently isolated from a single swine wastewater sample collected from a local farm. The water sample was centrifuged at 8,000×*g* for 10 min and then filtered through a 0.22 μm membrane filter to remove bacterial cells. The filtrate was enriched by mixing it with an early log-phase culture of the corresponding host bacterium and double-strength LB broth, then incubating at 37 °C with shaking for 12–18 h. After enrichment, the lysate was centrifuged and filtered. The presence of phages was confirmed using the spot test. Individual, well-isolated plaques were selected and purified at least thrice to ensure clonal purity.

### 2.3 Phage plaque and morphology characterization

Plaque morphology was assessed on double-layer agar plates after 18 h of incubation at 37 °C. The size, transparency, and presence of halos in at least 20 plaques were meticulously recorded. For transmission electron microscopy (TEM), purified phage suspensions were negatively stained with 2% (w/v) uranyl acetate. The grids were examined using a Hitachi HT-7700 TEM at 80 kV. Morphological classification was performed according to the guidelines established by the International Committee on Taxonomy of Viruses.

### 2.4 Host range analysis

The lytic spectrum of each phage was assessed against a panel of bacterial strains using a spot test. Briefly, 5 μL of a high-titer phage lysate (≥ 10^8^ PFU/mL) was applied to a lawn of the target bacteria. After incubation, the plates were examined for plaque formation and lysis.

### 2.5 Optimal multiplicity of infection (MOI)

The optimal MOI was determined by infecting mid-log-phase host cultures at various MOIs (10^−3^, 10^−2^, 10^−1^, 10^0^, 10^1^, 10^2^, and 10^3^). The phage-bacterial mixtures were incubated at 37 °C for 20 min to facilitate adsorption. The mixtures were then centrifuged to remove unadsorbed phages. The resulting pellets were re-suspended in fresh LB broth and incubated for 4–5 h. Following incubation, the lysates were centrifuged, and the supernatants were titrated. The MOI that produced the highest phage titer was considered optimal.

### 2.6 One-step growth curve

The incubation period and outbreak size were characterized using a one-step growth experiment. Briefly, when the MOI was set to 0.1, the phage-bacterial mixture was allowed to adsorb for 10 min. Subsequently, the mixture was centrifuged, re-suspended in fresh pre-heated broth, and incubated at 37 °C. Samples were collected every 10 min (at 0, 10, 20, 30, 40, 50, 60, 70, 80, and 90 min) for immediate titration. The incubation period was determined from the growth curve, and the outbreak size was quantified as the ratio of the final phage titer after the ascending phase to the initial number of infected bacteria.

### 2.7 Thermal stability

The thermal stability of the phages was evaluated by incubating 1 mL of phage suspension (approximately 10^8^ PFU/mL) in a water bath at 30, 40, 50, 60, 70, 80, and 90 °C. Aliquots were collected at 20, 40, and 60 min intervals, immediately cooled on ice, and subsequently titrated. The phage titer at each time point was expressed as a percentage of the initial value.

### 2.8 Determination of phage pH stability

The pH stability of the phages was assessed by incubating 1 mL of phage suspension (approximately 10^8^ PFU/mL) in a water bath at pH values ranging from 1 to 14. Aliquots were collected at 20-, 40-, and 60-min intervals and titrated. The phage titer at each time point was expressed as a percentage of the initial titer.

### 2.9 Genome sequencing, assembly, and annotation

Genomic DNA was extracted from purified high-titer phage lysates using a Phage DNA Isolation kit. Sequencing was performed using the Illumina NovaSeq 6,000 platform with a 150-bp paired-end approach. Raw reads were quality-trimmed and assembled into contiguous sequences using SPAdes software (version 3.15.5). Open reading frames were predicted using Prokka (version 2.6.1) or RAST (Brettin et al., 2015), and functional annotation was performed by searching the NCBI Non-redundant, Swiss-Prot, and conserved domain databases. tRNA genes were identified using tRNAscan-SE (version 2.0.9). Complete genome sequences were deposited in GenBank under accession numbers.

The conserved gene sequences of bacteriophages were selected for functional analysis using NCBI BLAST. The 20 most homologous bacteriophage sequences were identified and analyzed using MEGA7 software. Multiple sequence alignment was performed using Muscle, and the phylogenetic tree was constructed using the neighbor-joining method.

### 2.10 Phage and antibiotic combination test

The host strains, *E. coli* BYEC01 and *S. enterica* ATCC 14028, were cultured in Mueller-Hinton Broth at 37 °C with agitation at 180 rpm to test the phage-antibiotic synergistic effect. In this study, antibiotics were selected based on their sensitivity or resistance profiles: Polymyxin B (sensitive to *E. coli* BYEC01), Ampicillin (resistant to *E. coli* BYEC01 but sensitive to *S. enterica* ATCC 14028), and Sulfafurazole (resistant to *S. enterica* ATCC 14028). The preparation and inoculation of the culture medium were performed according to the K-B method, and the operational requirements were outlined in the EUCAST European Union drug-sensitivity testing standards.

### 2.11 Bactericidal efficacy of phages on the surfaces of milk and porcine skin

All food samples were confirmed to be free of the target pathogens before artificial contamination. The food samples were subsequently contaminated with suspensions of the target pathogens, resulting in final concentrations of approximately 10^5^ CFU/mL in both milk and pig skin. The contaminated samples were air-dried in a biosafety cabinet for 30 min to facilitate bacterial adherence to the surface. Subsequently, the samples were treated with a phage suspension in SM buffer to achieve final phage concentrations of 10^6^ and 10^7^ CFU/mL. The control group received an equivalent volume of sterile SM buffer. The processed milk and pig skin samples were stored at 4 °C and 25 °C, simulating common storage conditions. Samples were collected at predetermined time points (1, 3, 6, and 12 h) for bacterial enumeration. The high MOIs (1,000 and 10,000) were utilized in this initial proof-of-concept study to rigorously evaluate and demonstrate the maximum bactericidal potential of the phages under exaggerated conditions, rather than to reflect typical levels of natural contamination.

### 2.12 Mouse infection and phage therapy

Two-week-old Kunming mice, free of specific pathogens, were randomly divided into three groups (10 mice per group): (1) phage treatment, (2) infection control, and (3) simulated infection control (phosphate-buffered saline (PBS) treatment). The target pathogens, either 2 × 10^8^ CFU/mL of *E. coli* BYEC01 or *S. enterica* ATCC 14028, were, respectively, gavaged into the phage treatment and infection control groups. Two hours post-infection, the mice in the phage treatment group were administered 400 μL of phage solution at a concentration of 2 × 10^8^ CFU/mL via gavage, whereas those in the infection control group were administered an equivalent volume of sterile PBS.

The survival rate and weight changes of all the mice were recorded daily at consistent times. After 48 h of intragastric inoculation, five mice from each group were euthanized by cervical dislocation. Spleen and intestinal tissues were collected aseptically for further analysis.

### 2.13 Phage safety assessment

Twenty healthy Kunming mice, aged 2–3 weeks, were selected and randomly assigned to four groups (*n* = 5 per group). Mice in the experimental groups received an intraperitoneal injection of 0.2 mL of phage solution at a titer of 10^8^ PFU/mL, whereas the control group received an equal volume of sterile phosphate-buffered saline (PBS). All animals were maintained under identical environmental conditions. Seven days post-injection, the mice were euthanized by cervical dislocation, and organs were harvested for histopathological examination to assess potential pathological alterations.

### 2.14 Statistical analysis

The experimental data were statistically processed and expressed as mean ± standard deviation (SD). The difference between the experimental and control groups was determined using Student’s t-test, and the difference between the two experimental groups was determined using Tukey’s test for multiple comparisons using the Statistical Package for the Social Sciences software (version 17.0). Statistical significance was set at *p* < 0.05.

## 3 Results

### 3.1 Isolation and basic characterization of phages vB_EcoM_BYEP01 and vB_SalS_SP14

**Figure 1:**
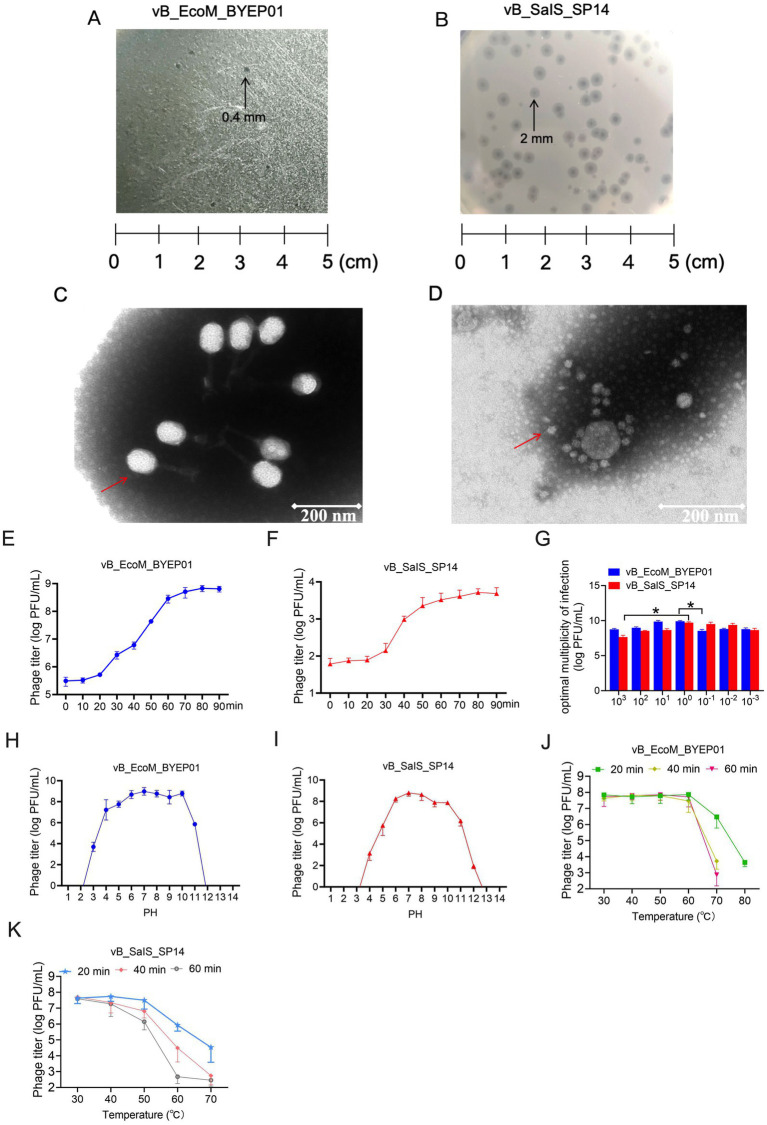
Isolation and basic characterization of phages vB_EcoM_BYEP01 and vB_SalS_SP14. **(A, B)** Plaque morphology of phage vB_EcoM_BYEP01 **(A)** and phage vB_SalS_SP14 **(B)** on a double-layer LB agar plate after 12 h of incubation at 37 °C. **(C, D)** TEM images of phages vB_EcoM_BYEP01 **(C)** and vB_SalS_SP14 **(D)**, with a scale bar of 200 nm. **(E, F)** Single-step growth curves for phages vB_EcoM_BYEP01 **(E)** and vB_SalS_SP14 **(F)**. The latent period and burst size were determined by infecting the host bacterium at an MOI of 0.1. **(G)** Determination of the optimal MOI: Host bacteria were infected at various MOIs, and the phage titer in the lysate was measured after completing the lytic cycle. **(H, I)** Stability assessment of phages vB_EcoM_BYEP01 **(H)** and vB_SalS_SP14 **(I)** under different pH conditions. pH stability was evaluated after incubating each phage in buffers with varying pH levels for 1 h, followed by measurement of the residual phage titer using the double-layer agar method. **(J, K)** The thermal stability of each phage was evaluated at different temperatures by incubating the phages at specified temperatures for 1 h before measuring residual titers using the double-layer agar method. All data are presented as mean ± SD (*n* = 3).

The two bacteriophages, vB_EcoM_BYEP01 and vB_SalS_SP14, were successfully isolated from a single swine wastewater sample using *E. coli* BYEP01 and *S. enterica* ATCC 14028 as host strains. Initial plating on LB agar revealed distinct plaque morphologies for all phages. Bacteriophage vB_EcoM_BYEP01 formed small (0.4 mm in diameter) and clear plaques, implying its potential ability to lyse bacteria (Figure 1A). Conversely, plaques produced by phage vB_SalS_SP14 were larger (2 mm) and clear, surrounded by a sharp halo (Figure 1B).

TEM was used to elucidate their structural differences further. Based on plaque morphology, TEM imaging confirmed that the two bacteriophages belonged to different families. Bacteriophage vB_EcoM_BYEP01 features an icosahedral head and a long, contractile tail, classifying it as a member of the *Myoviridae* family (Figure 1C). Meanwhile, vB_SalS_SP14 was characterized as an icosahedral structure devoid of a tail and exhibited features typical of the *Microviridae* family (Figure 1D). This morphological divergence was the first indication of distinct evolutionary lineages.

Subsequently, the lytic capabilities of the bacteriophages were quantitatively assessed by determining their one-step growth parameters and optimal MOI. The one-step growth curve revealed that bacteriophage vB_EcoM_BYEP01 exhibited a latent period of 10 min and a burst size of approximately 70 plaque-forming units (PFU) per infected cell (Figure 1E). Bacteriophage vB_SalS_SP14 exhibited a slightly longer latent period of 20 min and a comparable burst size of approximately 23 PFU/infected cell (Figure 1F). The optimal MOI was found to be 1 for both phages, yielding the highest progeny phage titer (Figure 1G). Notably, the maximum phage yield was achieved at an MOI of 1 for both phages, this observation aligns with the classical principle that an MOI favoring predominantly single infections minimizes inter-viral competition for host cellular resources and avoids premature lysis, thereby maximizing burst size and overall progeny production. These results indicate that, despite their structural divergence, both phages are efficient lytic agents with robust reproductive potentials.

Finally, the stability of the two bacteriophages was assessed at varying pH and temperature conditions. Both bacteriophages vB_EcoM_BYEP01 (Figure 1H) and vB_SalS_SP14 (Figure 1I) demonstrated robust tolerance to both acidic and basic conditions, exhibiting significant stability within a pH range of 6–10. The impact of temperature on bacteriophage vB_EcoM_BYEP01 is illustrated in Figure 1J. Within the temperature range of 30–60 °C, the phage titer remained relatively stable across different time points (Figure 1J). The impact of temperatures ranging from 30 to 50 °C on the bacteriophage vB_SalS_SP14 was minimal (Figure 1K).

In this study, we analyzed the biological characteristics of two newly isolated bacteriophages and revealed that both are lytic phages. The isolation and identification of novel lytic bacteriophages as alternatives to antibiotics can effectively eliminate bacteria. Furthermore, this study establishes a basis for improving the diversity of bacteriophage libraries and facilitates in-depth investigations into the functions of bacteriophage genes.

### 3.2 Host range of phages vB_EcoM_BYEP01 and vB_SaIS_SP14

To investigate the host range of the bacteriophages, 21 bacterial strains from various sources, along with five standard strains, were collected from regions including Guangxi, Guangdong, and Sichuan in China. The 22 *E. coli* strains comprised: (i) 21 environmental isolates obtained from the same porcine wastewater source as the phages, representing the local and contemporary bacterial population; and (ii) one reference strain (*E. coli* CVCC 1527) acquired from a national culture collection. Similarly, the four *Salmonella* strains were all reference type strains obtained from major international culture collections: CVCC 3384, CVCC 1806, ATCC 14028, and CMCC 50746. This selection intentionally included strains of diverse serotypes and geographic origins, enabling us to assess the efficacy of the phages against clinically relevant *Salmonella* types that extend beyond their primary host range, using well-characterized isolates. The findings revealed that the bacteriophages vB_EcoM_BYEP01 could lyse *E. coli* strains BYEC01, BYEC02, BYEC04, BYEC05, BYEC06, and BYEC07. Conversely, bacteriophage vB_SalS_SP14 exhibited the ability to lyse only *S. enterica* ATCC 14028 (Table 1).

Analysis of the phage lysis spectra demonstrated that the lysis profiles of the two phages isolated in this study were relatively narrow, indicating a high degree of host specificity.

### 3.3 Genomic and phylogenetic analysis of bacteriophages vB_EcoM_BYEP01 and vB_SalS_SP14

To elucidate the genetic basis underlying the distinct morphologies and shared lytic efficiency of vB_EcoM_BYEP01 and vB_SalS_SP14, we sequenced and analyzed their genomes.

**Figure 2:**
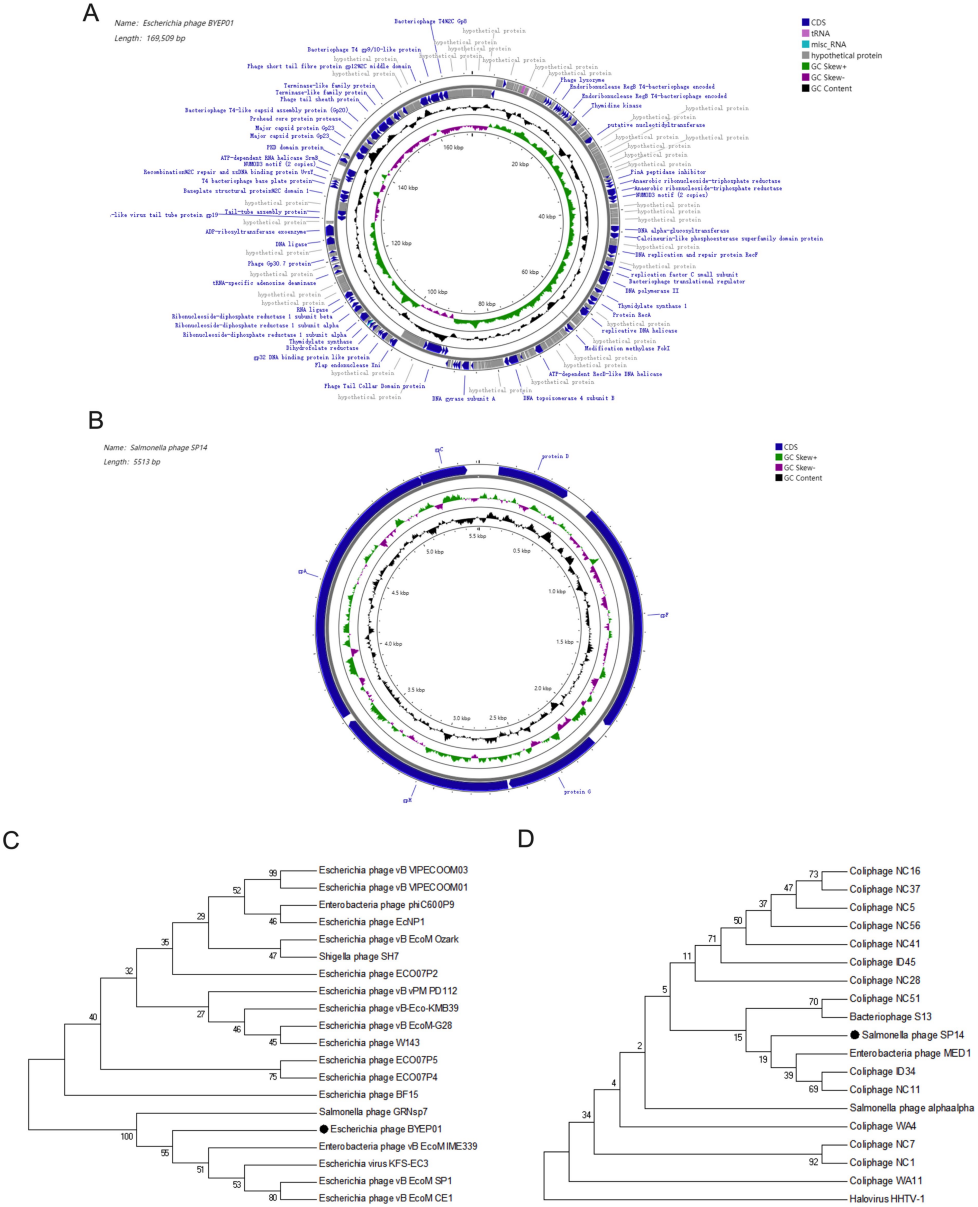
Genomic and phylogenetic analysis of bacteriophages vB_EcoM_BYEP01 and vB_SalS_SP14. **(A, B)** Circular genomic maps of vB_EcoM_BYEP01 **(A)** and vB_SalS_SP14 **(B)**. The outermost circle represents the coding regions (CDS) on both the positive and negative strands, whereas the innermost circle illustrates the G + C skew. **(C, D)** Phylogenetic analyses of vB_EcoM_BYEP01 **(C)** and vB_SalS_SP14 **(D)** were performed based on the sequences of their main capsid proteins. The bacteriophages examined in this study are represented by black dots.

The genome of vB_EcoM_BYEP01 was characterized as a double-stranded DNA molecule of 169,509 bp with a G + C content of 35.27%. Through analysis of the VFDB database, no toxin-related genes were identified in the genome of vB_EcoM_BYEP01 (Figure 2A). Conversely, the vB_SalS_SP14 genome was significantly smaller, consisting of a 5,513 bp single-stranded DNA molecule with a higher G + C content of 44.93%. Through analysis of the VFDB database, no toxin-related genes were identified in the genome of vB_SalS_SP14 (Figure 2B). Furthermore, tRNA scan identified seven tRNA genes within the vB_EcoM_BYEP01 genome, whereas no such genes were identified in the vB_SalS_SP14 genome. Further analysis revealed that no known antibiotic resistance genes were detected in the genomes of vB_EcoM_BYEP01 and vB_SalS_SP14 following comprehensive screening against the CARD and ResFinder databases (Figures 2A,B). These fundamental genomic features underscore the significant differences between the two phages. The bacteriophage vB_EcoM_BYEP01 data were successfully uploaded to the NCBI database with accession number OR757453.1. Furthermore, the bacteriophage vB_SalS_SP14 data were submitted to the NCBI database under the accession number PP501405.1.

Phylogenetic analyses were used to determine their evolutionary relationships. A phylogenetic tree constructed from the conserved major capsid protein placed vB_EcoM_BYEP01 within the *Tevenvirinae* subfamily of *Myoviridae* (Figure 2C), whereas vB_SalS_SP14 was within the *Bullavirinae* subfamily of *Microviridae* (Figure 2D). Consistent with this, a BLAST comparison of the complete genomes revealed no significant similarities between the two phages, conclusively classifying them into two separate genera that evolved from distinct lineages.

A comparative analysis of the two phage genomes highlights their distinct evolutionary paths. Phage vB_EcoM_BYEP01 harbors a large (~169 kbp), structurally complex genome characteristic of members of the *Myoviridae* family, with multiple genes organized into well-defined functional modules involved in DNA metabolism, virion assembly, and host cell lysis. In contrast, the genome of vB_SalS_SP14 is compact (~5.5 kbp) and streamlined, a feature commonly observed among *Microviridae*. Despite its reduced size, essential genes encoding the terminase (gpA), portal protein (gpH), major capsid protein (gpC), and tail components (protein D, gpF) are readily identifiable and exhibit conserved synteny (Figure 2B). This striking genomic divergence underscores the convergent evolution of lytic functionality from fundamentally different genetic architectures.

### 3.4 Enhancement of antibiotic efficacy against resistant pathogens through bacteriophage combination

**Figure 3:**
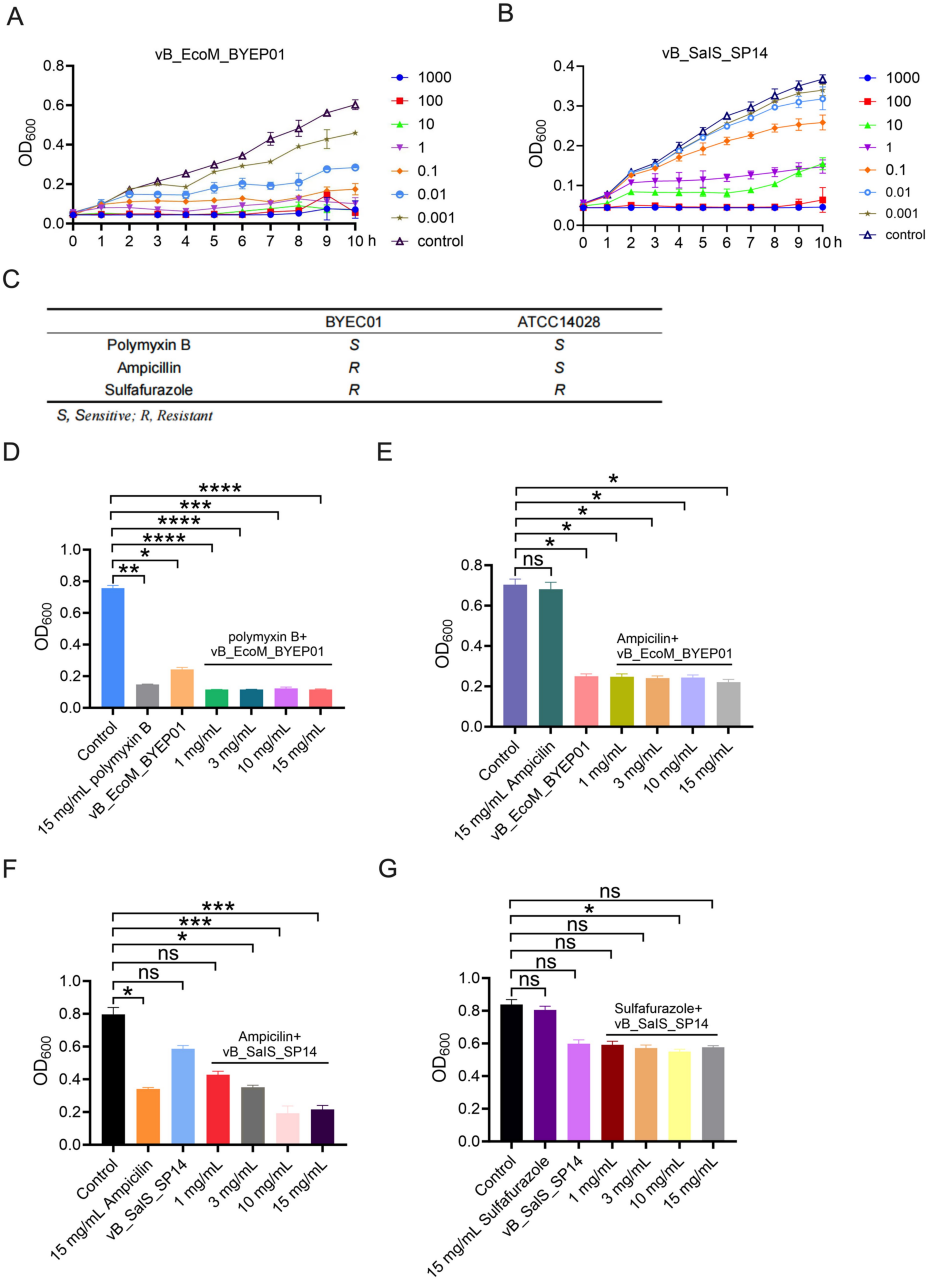
Enhancement of antibiotic efficacy against resistant pathogens through bacteriophage combination. **(A, B)** Lytic activity of phages vB_EcoM_BYEP01 **(A)** and vB_SalS_SP14 **(B)** at different MOIs against their host bacterium. Bacterial growth was assessed by measuring the optical density at 600 nm (OD_600_) over time. The phage-free group served as the control. **(C)** Analysis of host bacterial resistance. **(D)** The enhancement effect of phage vB_EcoM_BYEP01 in conjunction with the sensitive antibiotic Polymyxin B on host bacteria was evaluated. **(E)** The enhancement effect of phage vB_EcoM_BYEP01 in combination with the antibiotic Ampicillin, which exhibits drug resistance, on host bacteria was investigated. **(F)** The enhancement effect of phage vB_SalS_SP14 in conjunction with the sensitive antibiotic Ampicillin on host bacteria was evaluated. **(G)** The enhancement effect of the phage vB_SalS_SP14 in combination with the antibiotic Sulfafurazole, which exhibits drug resistance, on host bacteria was investigated. All data are presented as mean ± SD (*n* = 3).

Before performing experiments to evaluate the inhibitory effects of phages in combination with antibiotics on bacterial populations, we examined the *in vitro* lytic activities of the bacteriophages vB_EcoM_BYEP01 and vB_SalS_SP14 at various MOI. The bacteriophage vB_EcoM_BYEP01 effectively inhibited bacterial growth across an MOI range of 1–1,000, maintaining bacterial density at baseline levels throughout the experiment (Figure 3A). The lytic spectrum of bacteriophage vB_SalS_SP14 was also influenced by the MOI. Specifically, when the MOI ranged from 100 to 1,000, the phage demonstrated significant efficacy in inhibiting bacterial growth (Figure 3B).

After establishing the potent lytic efficacy of the individual phages *in vitro*, we investigated their potential for combination therapy with conventional antibiotics, a strategy aimed at improving antibacterial activity and mitigating resistance development. First, the antimicrobial resistance profile of the host bacteria was determined using the EUCAST European Antimicrobial Susceptibility Testing standard to evaluate the efficacy of the antibiotic control group against the target bacterial strain (Figure 3C). Subsequently, we used the antibacterial activity curve to assess the inhibitory efficacy of bacteriophages (vB_EcoM_BYEP01 or vB_SalS_SP14) against sensitive and resistant antibiotics in their respective host bacteria.

The antibacterial effect of bacteriophage vB_EcoM_BYEP01 in combination with Polymyxin B (a sensitive antibiotic) on *E. coli* BYEC01 is illustrated in Figure 3D. Compared to the control group, Polymyxin B and phage groups demonstrated significant inhibition of bacterial growth, with enhanced efficacy observed when antibiotics and phages were used together. There was minimal variation among the combination groups at different antibiotic concentrations (Figure 3D). The antibacterial effect of bacteriophage vB_EcoM_BYEP01 combined with Ampicillin (a resistant antibiotic) on *E. coli* BYEC01 is presented in Figure 3E. Compared to the control group, the Ampicillin group did not exhibit a significant bactericidal effect. Although the use of bacteriophage vB_EcoM_BYEP01 alone significantly suppressed bacterial growth, its combination with Ampicillin demonstrated a enhancement effect (Figure 3E). These findings indicate that bacteriophage vB_EcoM_BYEP01 exhibits a certain degree of enhancement activity with Ampicillin; however, its enhancement effect when combined with Polymyxin B was particularly significant.

The antibacterial effect of bacteriophage vB_SalS_SP14 in combination with Ampicillin (sensitive antibiotic) on *S. enterica* ATCC 14028 is illustrated in Figure 3F. Compared to the control group, Ampicillin and phage groups significantly suppressed bacterial growth, with a more pronounced effect observed when antibiotics and phages were used in conjunction (Figure 3F). The antibacterial effect of bacteriophage vB_SalS_SP14 combined with Sulfafurazole (resistant antibiotic) against *S. enterica* ATCC 14028 is depicted in Figure 3G. Compared to the control group, Sulfafurazole treatment did not exhibit a significant bactericidal effect. However, the combination of phages and antibiotics effectively inhibited bacterial growth, particularly at a concentration of 10 mg/L Sulfafurazole, which demonstrated superior efficacy (Figure 3G). These findings suggest that bacteriophage vB_SalS_SP14 exhibits a certain degree of enhancement activity with Sulfafurazole, whereas its enhancement effect with Ampicillin is even more pronounced.

These results clearly demonstrate that the bactericidal activity is significantly enhanced when the two bacteriophages are administered in combination with antibiotics at sublethal concentrations. This enhancement effect facilitates a more rapid and complete eradication of the bacterial population.

### 3.5 Biocontrol potential in a food model system

To evaluate the potential of vB_EcoM_BYEP01 and vB_SalS_SP14 as biological control agents for practical use, we investigated their inhibitory effects on the host bacteria in contaminated food samples. Milk and pig skin were selected as representative models for the liquid and solid food substrates, respectively. The food samples were inoculated with their corresponding host bacteria and treated with two doses of the respective phage 10^6^ PFU/mL (MOI = 1,000) and 10^7^ PFU/mL (MOI = 10,000). The samples were then stored at 4 °C (refrigerated) and 25 °C (room temperature) to simulate common storage conditions.

**Figure 4:**
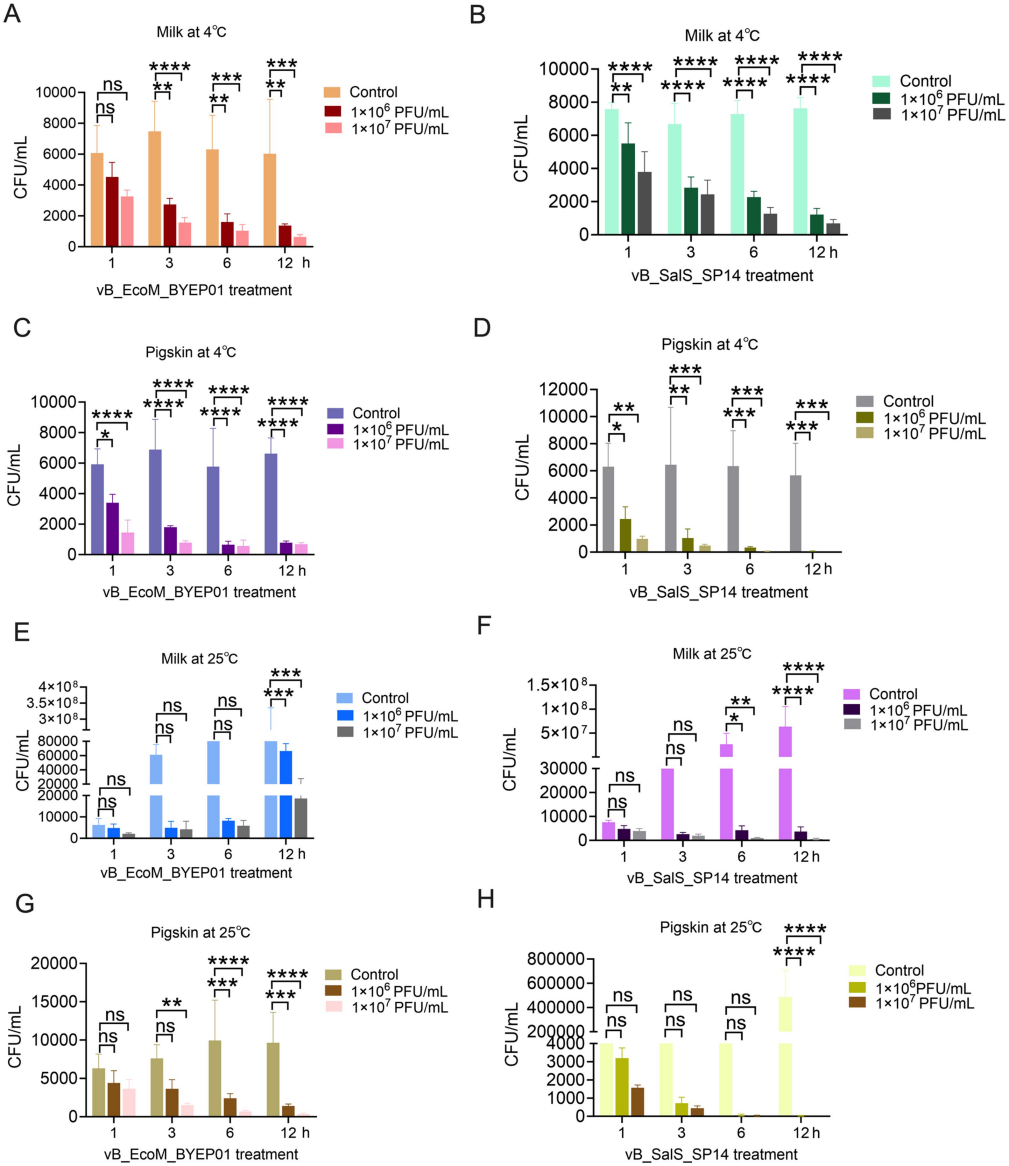
Biocontrol potential in a food model system. **(A–D)** The inhibitory efficacy of various doses of vB_EcoM_BYEP01 **(A, C)** and vB_SalS_SP14 **(B, D)** against their respective host bacteria under storage conditions of 4 °C milk and pig skin. **(E–H)** The inhibitory efficacy of various doses of vB_EcoM_BYEP01 **(E, G)** and vB_SalS_SP14 **(F, H)** phages against their respective host bacteria under storage conditions of 25 °C milk and pig skin. All data are presented as mean ± SD (*n* = 3).

At 4 °C, phage therapy demonstrated significant and sustained antibacterial activity. In milk, compared to the untreated control group, a dose of 1,000 MOI vB_EcoM_BYEP01 reduced the viable *E. coli* count by 0.96 orders of magnitude after 12 h, whereas a dose of 10,000 MOI vB_EcoM_BYEP01 resulted in a reduction of 1.3 orders of magnitude after the same duration (Figure 4A). Similarly, at 4 °C in milk, the administration of 1,000 MOI of vB_SalS_SP14 resulted in a decrease in viable *Salmonella* counts by 0.8 orders of magnitude, whereas at a concentration of 10,000 MOI, vB_SalS_SP14 achieved a reduction of 1.04 orders of magnitude. Bacterial growth was effectively inhibited throughout the entire course of the experiment (Figure 4B). Furthermore, no significant change was observed in the number of live bacteria on pig skin stored at 4 °C within the control group. Conversely, a persistent decrease in viable bacteria was observed in the vB_EcoM_BYEP01 treatment group (Figure 4C). Similarly, treatment with vB_SalS_SP14 on pig skin at this temperature demonstrated significant antibacterial efficacy (Figure 4D).

Lower temperatures seem to decelerate both bacterial metabolism and phage replication. However, phages effectively impede pathogen proliferation during refrigerated storage, highlighting their efficacy as protective agents.

More dynamic and rapid bactericidal effects were observed at 25 °C. In milk, the use of 10,000 MOI vB_EcoM_BYEP01 significantly suppressed *E. coli* activity within 12 h (Figure 4E). Similarly, treatment with 10,000 MOI vB_SalS_SP14 resulted in a significant reduction in *Salmonella* activity after 12 h (Figure 4F). For *E. coli* on pig skin, vB_EcoM_BYEP01 also significantly inhibited bacterial activity (Figure 4G). Similarly, for *Salmonella* on pig skin, treatment with vB_SalS_SP14 resulted in a significant decrease in *Salmonella* activity within 12 h, maintaining a significantly lower bacterial load than the rapidly increasing control group (Figure 4H). At this environmental temperature, the enhanced efficacy may be attributed to active phage replication and an accelerated lysis cycle that effectively surpasses the bacterial proliferation.

Collectively, these findings underscore the practical significance of both phages as promising biocontrol agents. They effectively limit the survival and growth of target pathogens across different food types and at clinically relevant storage temperatures, providing a targeted strategy for improving food safety.

### 3.6 Phage therapy confers full protection and significantly reduces bacterial burden in a murine infection model

**Figure 5:**
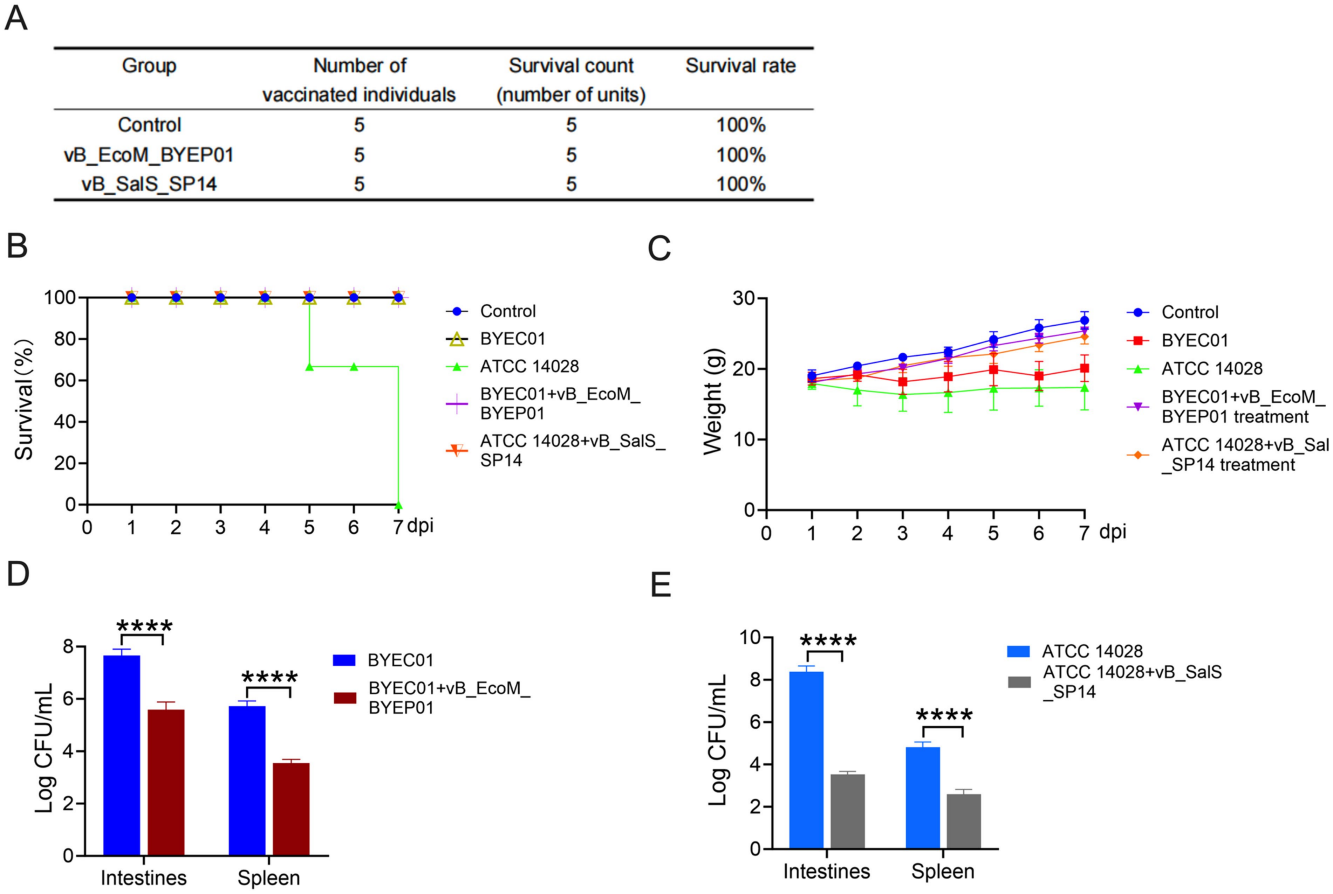
Phage therapy significantly reduced bacterial levels in the murine infection model. **(A)** Phage safety testing. **(B)** Survival rate of mice. **(C)** Fluctuations in the weight of mice. **(D, E)** Bacterial concentration in murine organs. All data are presented as mean ± SD (*n* = 5).

Before establishing the *in vitro* infection model, we assessed the safety of the two phages. Phage solutions were administered intraperitoneally to mice over a period of 7 days. No adverse reactions were observed in any of the animals, and postmortem examinations revealed no tissue or organ lesions. These findings indicate that the two phages exert no toxic effects in mice. The mouse survival rate is presented in Figure 5A.

The therapeutic potential of vB_EcoM_BYEP01 and vB_SalS_SP14 was assessed *in vivo* using mouse infection models. The lethal doses of each pathogen were administered to the mice via gavage. Two hours post-infection, the corresponding phage (1 × 10^9^ PFU) or PBS was administered by gavage for control purposes.

The phage therapy experiment in mice was meticulously monitored for 7 days. The survival outcomes were unambiguous. The survival rate in the *E. coli* infection group was 100%, with no fatalities in the mice. Conversely, the *Salmonella* infection group exhibited a survival rate of 60%, whereas no deaths occurred in the treatment group (Figure 5B). Furthermore, there was no significant decrease in body weight within the phage treatment group, indicating robust treatment tolerance and effective prevention of systemic deterioration in control animals (Figure 5C).

To quantitatively evaluate the bactericidal efficacy of bacteriophages *in vivo*, we measured the bacterial load in the primary target organs 48 h post-infection. In the control group, a significantly high level of bacterial colonization was observed in the spleens and intestines. Conversely, phage therapy significantly reduced bacterial burden. In the vB_EcoM_BYEP01 treatment group, the *E. coli* load in both the spleen and intestine decreased by 2.09 and 2.13 orders of magnitude, respectively, compared to the control group (Figure 5D). Similarly, treatment with vB_SalS_SP14 resulted in a reduction of 1.02 orders of magnitude for *Salmonella* in the spleen and a significant decrease of 3.32 orders of magnitude for *Salmonella* in the intestine (Figure 5E). These findings indicate that bacterial counts in the phage treatment group approached or fell below the detection limit, thereby confirming the effective clearance of pathogens from the systemic circulation and major organs.

Collectively, these *in vivo* findings demonstrate that a single dose of either vB_EcoM_BYEP01 or vB_SalS_SP14 is safe and significantly effective, achieving 100% survival by rapidly controlling and clearing lethal bacterial infections in a systemic model.

## 4 Discussion

*Escherichia coli* and *Salmonella* are Gram-negative opportunistic pathogenic bacteria that are widely distributed in nature and have resulted in significant economic losses globally. These pathogens primarily manifest clinical symptoms such as diarrhea, vomiting, fever, and sepsis and can infect humans through different routes (Gomes et al., 2016; Biran and Ron, 2018; Ferrari et al., 2019; Christenson, 2013). Recently, bacterial resistance has increased due to the overuse of antibiotics and intensive farming practices (Paitan, 2018; Kim et al., 2017; Gut et al., 2018). Consequently, many new classes of antibiotics have lost their efficacy, rendering the prevention and control of bacterial diseases increasingly challenging. Numerous industries are actively pursuing novel formulations as alternatives to antibiotics for preventing and treating bacterial infections. Phages, recognized as natural predators of bacteria, have recently garnered significant attention from researchers both domestically and internationally because of their unique antibacterial properties (Monteiro et al., 2019; Bao et al., 2019; Malik et al., 2017; Zyman et al., 2022; Danis-Wlodarczyk et al., 2021).

In this study, we successfully isolated and characterized two bacteriophages: vB_EcoM_BYEP01 and vB_SalS_SP14. Bacteriophage vB_EcoM_BYEP01 features an icosahedral head with a retractable tail and is classified in the *Myoviridae* family. Conversely, bacteriophage vB_SalS_SP14 exhibited an icosahedron without any tail structure and belonged to the *Microviridae* family (Figure 1). Furthermore, the cleavage spectra of the two phages isolated in this study were relatively narrow, indicating a high degree of host specificity (Table 1). However, owing to the influence of the varieties and quantities of bacteria preserved by the scientific research institution, it cannot be conclusively determined that these bacteriophages are narrow-spectrum until extensive bacterial lysis experiments have been performed. Although they can lyse a limited number of bacterial strains, further investigations are needed to establish their spectrum definitively. Furthermore, studying bacteriophage genomes serves as a foundation for modifying these structures. In this study, the genomic data for bacteriophage vB_EcoM_BYEP01 indicated that its coding genes totaled 159,899 bp, with a G + C content of 35.27%. In comparison, the coding gene length of bacteriophage vB_SalS_SP14 was 5,037 bp, exhibiting a G + C content of 44.93% (Figure 2).

In addition to their fundamental biology, the practical use of these phages is strongly corroborated by our functional data. Bacteriophage vB_EcoM_BYEP01 demonstrated a significant enhancement effect when combined with either the sensitive antibiotic Polymyxin B or the drug-resistant antibiotic Ampicillin. Similarly, bacteriophage vB_SalS_SP14 exhibited a significant enhancement effect when combined with the sensitive antibiotic Ampicillin or the drug-resistant antibiotic Sulfafurazole (Figure 3). This synergism suggests that our bacteriophages can be effectively integrated into existing treatment regimens to enhance the efficacy of traditional antibiotics, thereby providing a robust combinatorial strategy for addressing multidrug-resistant infections.

The translational potential of bacteriophages vB_EcoM_BYEP01 and vB_SalS_SP14 extends from clinical applications to food safety. Their efficacy in reducing bacterial loads in complex food matrices, such as milk and pork skin, under both refrigeration and ambient temperatures highlights their robustness as biocontrol agents (Figure 4). The ability to maintain efficacy under common storage temperatures is critical for phages. This positions vB_EcoM_BYEP01 and vB_SalS_SP14 as natural, targeted preservatives capable of mitigating the risk of foodborne illnesses caused by *E. coli* and *S. enterica*, respectively. Future studies will assess the efficacy of this phage cocktail across a broader range of food products, including chicken and leafy vegetables.

The most compelling evidence for the therapeutic potential of our phages comes from an *in vivo* murine model. Neither vB_EcoM_BYEP01 nor vB_SalS_SP14 exhibited toxicity or adverse effects in animal studies. Following intragastric inoculation of the host bacteria and subsequent treatment with phages, both demonstrated significant therapeutic efficacy (Figure 5). A limitation of this study is the absence of direct quantification of phage titers in target organs, which would be necessary to confirm their in vivo colonization and replication. Future studies will focus on assessing phage biodistribution and pharmacokinetics to gain a comprehensive understanding of the dynamic interactions between phages and pathogens at the site of infection.

The intragastric administration model utilized in this study provides valuable reference evidence for the potential clinical application of phage therapy in treating systemic bacterial infections. The significant reduction in bacterial loads observed in key organs following a single dose of phage administered shortly after infection highlights its potent in vivo activity. However, it is important to recognize the limitations of this model in fully recapitulating human clinical conditions. First, our model employed a single, genetically homogeneous bacterial strain, whereas clinical infections often involve heterogeneous or multidrug-resistant isolates, necessitating the use of broad-host-range phage cocktails to ensure therapeutic efficacy. Second, the immune status of healthy, immunocompetent mice differs substantially from that of typical clinical patients, who are frequently immunocompromised, elderly, or afflicted with comorbidities—factors that may profoundly influence phage pharmacokinetics, biodistribution, and overall treatment outcomes.

In conclusion, our research extends beyond the identification of two novel bacteriophages. Their demonstrated efficacy in overcoming antibiotic resistance *in vitro*, ensuring food safety, and protecting animals from life-threatening infections underscores their potential. Future research should focus on evaluating the efficacy of a specific phage cocktail against co-infections and optimizing the administration formulations for clinical and industrial applications.

## Data Availability

The datasets presented in this study can be found in online repositories. The names of the repository/repositories and accession number(s) can be found in this article. The GenBank accession number for the phage vB_EcoM_BYEP01 whole genome is OR757453.1. The GenBank accession number for the phage vB_SalS_SP14 whole genome is PP501405.1.
